# Assessing wellbeing in foundation doctors during the COVID-19 pandemic

**DOI:** 10.1192/bjo.2021.513

**Published:** 2021-06-18

**Authors:** Nikhita Handa, Sanjeev Pramanik

**Affiliations:** East Lancashire Hospitals NHS Trust

## Abstract

**Aims:**

The COVID-19 pandemic has had a drastic effect on the mental health of the global population that is likely to be felt for years to come. One group particuarly likely to be affected by this in the immediate future are the healthcare professionals working on the frontline of the NHS pandemic response. As members of a foundation cohort of these junior doctors we aimed to create a way to quanitfy the wellbeing of ourselves and our colleagues at this challeging time. We aimed to use a combination of numerous tools to monitor foundation doctors in Blackburn during this crisis. This would inform which measures would be best suited to be put in place to protect this cohort from early burnout and poor mental health in the future.

**Method:**

We designed a survey of 25 questions which we invited our foundation colleagues to fill in anonymously during the first and second waves of the pandemic in response to times when foundation doctors were redeployed to aid the frontline. The survey has been based on the PHQ9, GAD7, Epworth Sleepiness scale, Physician wellbeing index, Medical students wellbeing index, Maslach burnout inventory BMA burnout questionnaire and the QOL scale.

**Result:**

From a cohort of around 140 foundation doctors we had 46 participants in our trial of this tool; 46% had been redeployed and 54% not redeployed. Over 50% of survey respondents reported high stress, poor motivation and depersonalisation over the two weeks at the peak of the pandemic, key early signs of burnout. Lack of interest in their work, poor sleep and anhedonia were increased across both groups (redeployed and non redeployed). The interventions after the first wave data which repondents found beneficial included; financial reassurances during redeployments, protected non clinical areas for rest, a named individual senior staff member for wellbeing support.

**Conclusion:**

Key issues the survey raised were fed back to foundation programme leads in monthly meetings. This allowed us with our foundation leads to make targeted changes in order to support foundation doctors at this time. Without the data from this tool which we tailored to the foundation experience we believe these rapidly worsening issues during the pandemic would not have been addressed so swiftly. We then resurveyed the foundation cohort to assess which of these interventions have been most widely used and appreciated.

